# Is brace necessary after cervical surgery: A meta-analysis of randomized controlled trials

**DOI:** 10.1097/MD.0000000000029791

**Published:** 2022-07-08

**Authors:** Yang Mao, Zhao Jindong, Fang Zhaohui

**Affiliations:** a The First Affiliated Hospital of Anhui University of Traditional Chinese Medicine, Hefei, China; b Anhui University of Traditional Chinese Medicine, Hefei, China; c Diabetes Institute, Anhui Academy of Chinese Medicine, Hefei, China; d Key Laboratory of Xin'an Medicine, Ministry of Education, Hefei, China

**Keywords:** cervical brace, meta-analysis, postoperative cervical spine

## Abstract

**Background:**

Currently, there are increasing surgical treatments for neck pain. However, whether to use cervical brace after operation remains poorly defined. We aim to clear the clinical efficacy of the use of cervical brace after cervical surgery.

**Methods:**

We searched for relevant studies in 8 electronic databases up to March 2021. The mean difference and 95% confidence intervals were used for continuous data. Cochrane Collaboration’s tool was used to assess the risk of bias. The data were collected and input into the Review Manager 5.3 software (The Cochrane Collaboration, Copenhagen, Denmark).

**Results:**

Four randomized controlled trials were finally included in our study. For pain, the pooled analysis showed that postoperative neck brace compared with no brace can relieve neck pain at all follow-up periods except 6 months. For neck disability index, the result showed that postoperative neck brace compared with no brace can improve neck disability index during the 3 to 12 month follow-up period. However, no significant difference was identified between 2 groups within the follow-up of 6 weeks after surgery. In addition, the result tends to get the opposite at follow-up of 24 months. For 36-Short form health survey Physical Component Summary, there was no significant difference between 2 groups in the early 3 weeks after surgery, but the results were changed after 3 weeks. For 36-short form health survey Mental Component Summary, there appears to be no significant change between 2 groups at all time intervals.

**Conclusion:**

Wearing a cervical brace after cervical surgery is conducive to improving symptoms after cervical surgery at different stages. However, there is no relevant evidence indicating it can improve the mental health of postoperative patients. Higher quality, large prospective randomized studies are needed to verify the current conclusions.

## 1. Introduction

Neck pain, causing pain and disability, is a common condition in the present climate.^[1]^ As ranked 19th overall in the global cause of disability-adjusted life years, it has brought a great economic burden to society.^[1,2]^ According to the Global Burden of Disease 2010 Study, neck pain is the fourth greatest contributor to global disability, ranking behind back pain, depression, and arthralgias.^[3]^ Treatment options for neck pain include conservative and surgical measures. In addition, conservative treatment is a reasonable treatment choice for nonneuropathic neck pain.^[4]^ While surgical management can be beneficial to patients with cervical radiculopathy or myelopathy that had failed a course of nonoperative methods.^[5]^ Numerous studies^[6–9]^ have shown that cervical surgery can effectively alleviate the pain and symptoms of patients and improve functional activities. However, about 22% of patients will have postoperative complications such as loss of height of the intervertebral space, displacement of the implant, and decreased fusion rate.^[10–12]^ Hence, the cervical brace is often used after cervical surgery to improve the above complications.^[11,13,14]^

The use of cervical brace during postoperative period is commonly based on the belief that it may include the restriction of neck movement, providing spinal stability, reducing pain, and even increasing sense of security for patients.^[14–18]^ However, there is still no consensus regarding the use of cervical brace after cervical spine surgery. Several studies^[14,19]^ reported that cervical brace treatment was recommended for postoperative patients of cervical spine because of better relief of symptoms. Nevertheless, some studies^[20,21]^ suggested the postoperative treatment with brace immobilization was not inferior to those without brace immobilization. In addition, to the best of our knowledge, no clinical guidelines concerning the use of cervical brace after cervical spine surgery have been found.

The controversy surrounding the efficacy of cervical brace after surgery demonstrates the need for further exploration of the effects of cervical brace use following surgery. There are no published quantitative meta-analyses, which are often used to provide scientific guidance for clinical practice, investigating if the use of cervical brace actually improves postoperative clinical outcomes. Therefore, this meta-analysis was to evaluate the existing literature about the use of postoperative cervical brace to determine the strength of evidence.

## 2. Materials and methods

This article was performed in accordance with the Preferred Reporting Items for Systematic Reviews and Meta-analyses statement.^[22]^ Besides, it was based on previously conducted studies. Thus, no ethical approval and patient consent are required. The review protocol was registered with the International Prospective Register of Systematic Reviews registration, available online: http://www.crd.york.ac.uk/PROSPERO/display_record.php?ID=CRD42019121778.

### 2.1. Search strategy and selection criteria

As with the original review, we used the search strategies recommended by the Cochrane Back Review Group for the identification of randomized controlled trials (RCTs).^[23]^ An independent review of the Pubmed, Embase, Cochrane Library, Web of Science, Chinese National Knowledge Infrastructure Database, Wanfang database, China Biology Medicine, and VIP database was performed from inception to March 2021. There were no limits on study dates or any language, publication type, and status restrictions. The search was conducted with the following keywords: cervical surgery (replacement, prosthesis, fusion, decompression, discectomy, laminoplasty, ablation, and endoscope) and cervical orthoses (cervical gear, cervical collar, cervical splints, cervical support, cervical brace, cervical bracing, cervical orthoses, and cervical orthosis). Different search strategies were used for Chinese and English language databases. To identify trials that may not have been published in full or were missed through the electronic search, we manually searched all references from the included studies and relevant previous systematic reviews.

The retrieved literature was screened by 2 independent investigators to evaluate eligibility, and any discrepancies were settled by discussion and consensus. Studies that met the following criteria were included in the analysis: Patients: suffering from cervical surgeries, including anterior cervical discectomy and fusion, cervical laminoplasty, cervical disc replacement, cervical decompression, cervical endoscope, etc.; Intervention: external cervical immobilization; Control: without external cervical immobilization; Primary outcome: neck pain; and Study style: prospective RCTs. The excluded studies were excluded due to the following reasons: studies do not conform to the above criteria; studies were in the form of letters, abstracts, reviews, or comments; and studies were impossible to extract relevant data.

### 2.2. Data extraction

Two reviewers independently extracted several data using a predesigned data extraction form, and the results were compared to avoid bias from the data extraction process. The following characteristic information was extracted from each study: design (the name of the first author, year of publication, and country), participants (sample size, age, gender of patients, disease, and surgical method), interventions (external cervical brace type, and intervention period), and outcomes (neck pain, neck disability index [NDI], and 36-Short form health survey [SF-36]). When relevant data had not been reported, we contacted the authors by email or in other ways to attempt to obtain the missing information.

### 2.3. Assessment of risk of bias

We assessed the study quality of each included trial according to the Cochrane risk of bias tool for randomized trials.^[24]^ We assessed the following items: the generation for random sequence, concealment for allocation sequence, blinding of participants, incomplete outcome data, selective outcome reporting, and other sources of bias. For each included study, each type of bias was rated as high, low, or unclear and entered into the risk of bias table. The risk of bias was examined by 2 reviewers concurrently, and discrepancies were resolved by consensus.

### 2.4. Outcome measures

The 3 most recommended outcome indicators, which are Visual Analog Scale; NDI, 36-Short form health survey (SF-36), or 12-Short form health survey, in the guidelines on cervical radiculopathy issued by the North American Spine Society were selected as outcome indicators. In addition, the pain was the main outcome, and the secondary outcomes were NDI, SF-36, or 12-Short form health survey.

### 2.5. Grading the quality of evidence

We used the Grading of Recommendations Assessment, Development, and Evaluation (GRADE) (McMaster University, 2015) method to assess the quality of the evidence for each outcome of meta-analysis. Levels of quality of evidence were defined as high(++++), moderate(+++), low(++), and very low(+).^[25]^ We took into account the following items: risk of bias, inconsistency, indirectness, imprecision, and publication bias, and we operated on this web page: https://gradepro.org/.

### 2.6. Data synthesis and statistical analysis

We used DerSimonian and Laird random effects models in RevMan 5.3 (Nordic Cochrane Centre, Rigshospitalet, København, Denmark) to conduct the meta-analyses. The outcomes of interest only include continuous variables. We used mean difference (MD) to assess the difference in the continuous outcomes between the groups. Study weights were generated using the inverse of the variance. We present results as MD and associated 95% confidence intervals (CIs). In order to evaluate the sensibility of the meta-analysis, we excluded trials at high risk of bias. The significance threshold was a 2-sided *P* < 0.05. The forest plot for each parameter was constructed to illustrate the weight ratio of each incorporated study to evaluate potential publication bias if enough studies are included. We also used the GRADE approach to rate the quality of evidence and generate absolute estimates of effect for the outcome.^[26]^ Furthermore, the within-subject change standard deviation, which was calculated based on means and standard deviations at baseline and follow-up provided in articles, was required for the meta analysis.^[25]^ Thus, weighted by the sample size of each trial:


$$\begin{array}{ll} SD_{(\;follow-up-baseline)} & =\sqrt{S{D^{2}}_{baseline}+}S{D^{2}}_{follow-up}\\ & -(2\times Cor_{(\;follow-up-baseline)}\\ & \times SD_{baseline}\times SD_{follow-up}) \end{array}$$

## 3. Results

### 3.1. Literature search and study sample characteristics

**Figure 1.**
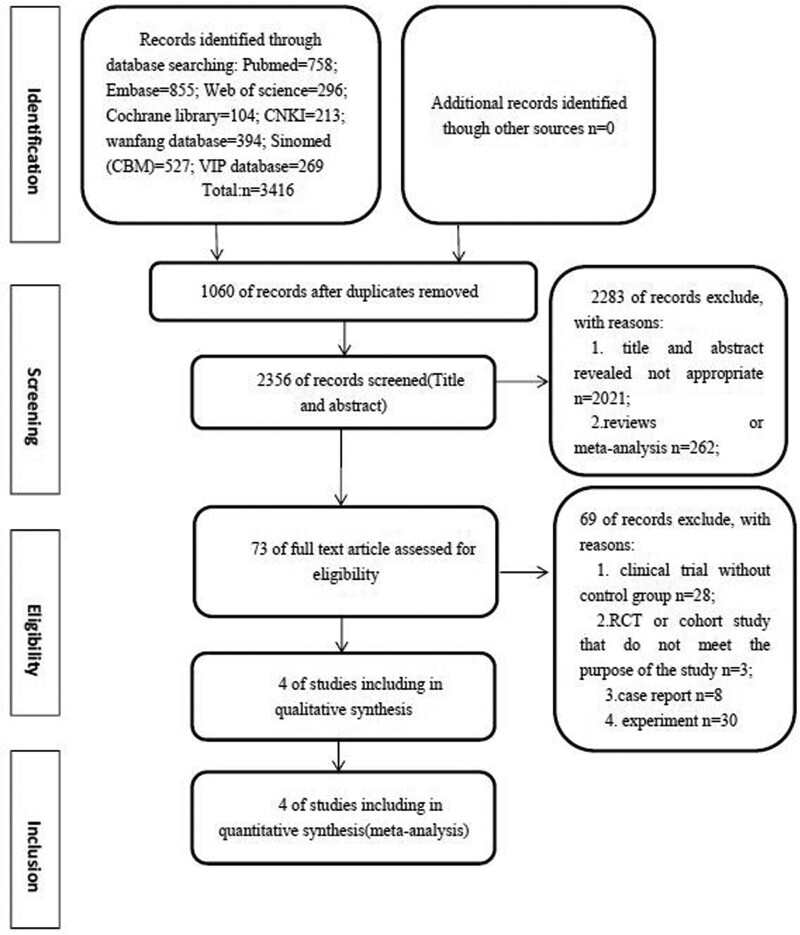
Flow diagram of the process of identifying relevant studies. CBM = China Biology Medicine, CNKI = Chinese National Knowledge Infrastructure, RCT = randomized controlled trial.

The search results are displayed in Figure 1. Our search of electronic databases retrieved 3416 records, of which 1060 were duplicates. According to the inclusion and exclusion criteria, 2283 articles were excluded after reading the title and summaries, containing 262 reviews or meta-analyses. We assessed 73 full-text articles, of which 4 were eligible.^[14,20,21,27]^ Among the excluded articles, 28 trials were excluded because these are clinical trials lack a control group, 3 studies were excluded because of RCTs or cohort studies that do not meet the purpose of this study, 8 case reports were excluded, and 30 studies were excluded because these belong to the field of basic researches. Table 1 presents the characteristics of the 4 eligible trials involving 208 participants (103 and 105 in the brace group and without brace group, respectively). The trial sample size ranged from 33 to 90 participants. All studies were compared with brace and without brace, 2 of which used rigid brace,^[14,27]^ 1 for semi-rigid brace,^[21]^ and 1 did not describe the brace used.^[20]^ The intervention period is reported between 2 and 6 weeks, of which 2 articles reported 6 weeks with brace after cervical surgery,^[14,27]^ 1 reported 3 weeks,^[27]^ and 1 reported 2 weeks.^[20]^ Two studies used brace for cervical myelopathy undergoing open-door laminoplasty,^[20,27]^ and 2 other studies used brace after anterior cervical discectomy and fusion. Baseline imbalance was not found in the demographic characteristics or the outcomes between the study groups. Three trials^[20,21,27]^ reported details of sample size calculations.

**Table 1:** Basic characteristics of the included trials.

Study ID	Sample size	Age (yr)	Cervical collar type	Time to immobilization	Intervention	Follow-up	Main outcomes
	Brace/No-brace (M/F)	Brace/No-brace					
Cheung 2018^[27]^	16 (10/6)/19(10/9)	61.7 ± 14.3/67.2 ± 8.4	Rigid cervical (Philadelphia collar)	3 wk	Open door laminoplasty	1 yr	VAS, SF-36, and NDI
China						1, 2, 3, and 6 wk and at 3, 6, and 12 mo postoperatively	
Overley 2018^[21]^	25/25	55.2 ± 11.72/50.15 ± 9.79	Semirigid cervical orthosis (Miami J cervical orthosis)	6 wk	Instrumented ACDF	1 yr	NDI
United States						2 and 6 wk and at 3, 6, and 12 mo postoperatively	
Hida 2017^[20]^	45(33/12)/45(28/17)	72.0 ± 8.7/71.6 ± 9.6	NA	2 wk	Modified double-door laminoplasty	1 yr	VAS and SF-36
						2 wk and at 3, 6, and 12 mo postoperatively	
Japan							
Abbott 2012^[14]^	17(9/8)/16(11/5)	53.4 ± 13/47.3 ± 11	Rigid cervical collar (Philadelphia Collar and Camp Scandinavia AB)	6 wk	ACDF	2 yr	NDI, SF-36, and VAS
Sweden						6 wk and at 3, 6, 12 and 24 mo postoperatively	

ID = identification; NA = not applicable; NDI = neck disability index, SF-36 = 36-short form health survey, VAS = Visual Analog Scale.

### 3.2. Risk of bias

**Figure 2.**
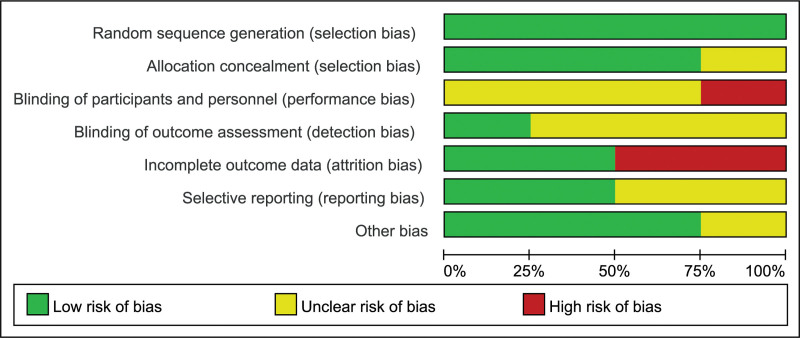
Risk of bias graph.

Figure 2 summarizes the graph of methodological quality. All the included studies described using the computer random method. Three studies showed allocation concealment using “envelope method.”^[14,20,27]^ And Overley et al’s^[21]^ study did not report it clearly. All studies^[14,20,21,27]^ either did not use blind methods or did not report it clearly. Only the study by Cheung et al^[27]^ described using blinding outcome assessment. Three trials^[14,20,27]^ reported participant losses during follow up period. In addition, the study by Cheung et al^[27]^ reported all the participants took part in the follow-up.^[27]^ Selective reporting was difficult to assess in 2 articles^[20,21]^ because trial protocols were unavailable. All judgments concluded low risk of bias (including incomplete outcome data, no blinding method, and loss to follow-up), suggesting future studies might influence the results in this meta-analysis and require revising the conclusion.

### 3.3. Meta-analysis outcome

#### 3.3.1. Cervical pain.

**Figure 3.**
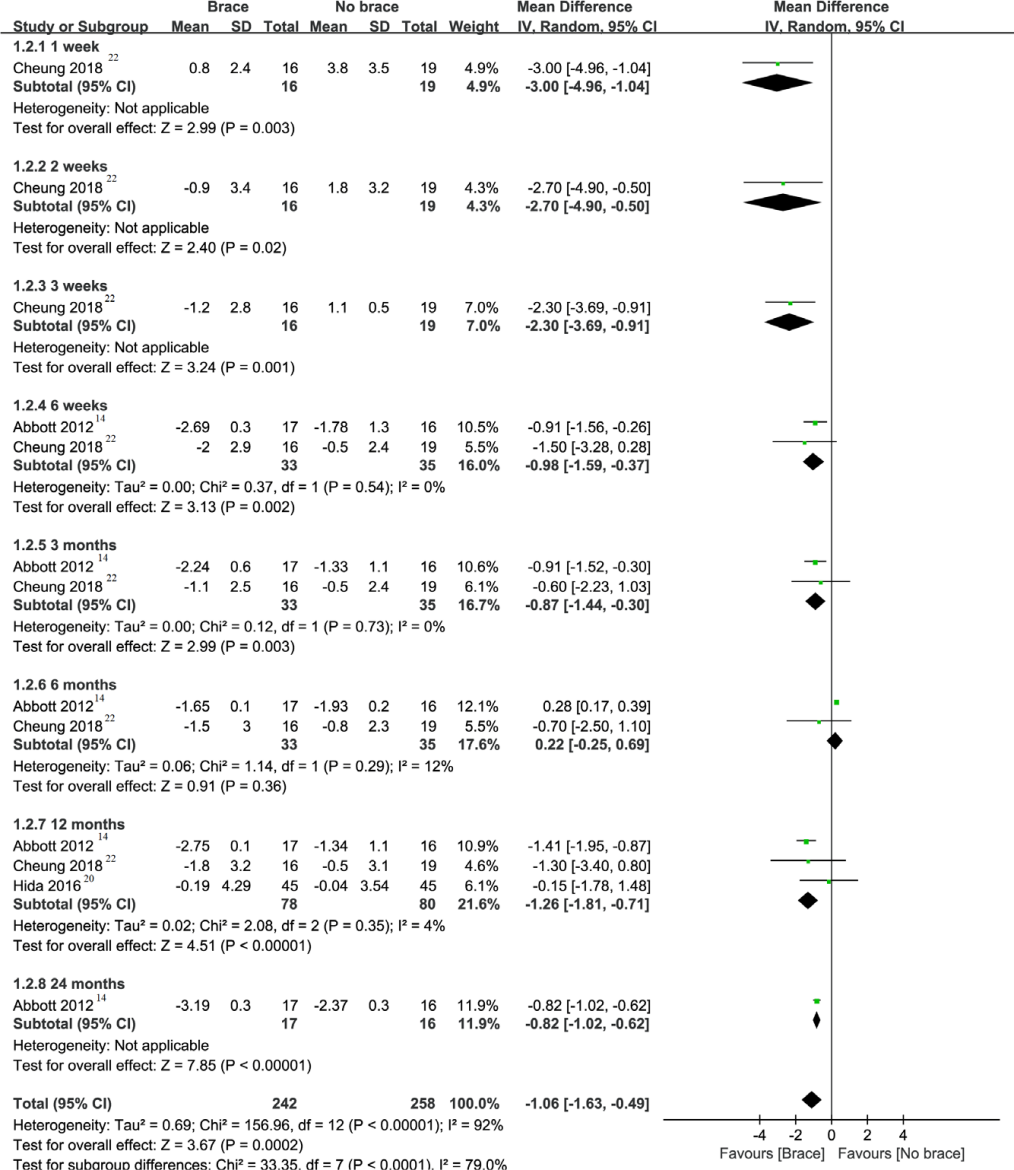
Forest plot and meta-analysis of cervical pain. CI = confidence interval, IV = inverse variance, SD = standard deviation.

The cervical pain is shown in Figure 3. Three studies including 158 patients^[14,20,27]^ reported the result of cervical pain. We performed a subgroup analysis according to different follow-up periods. Pooled analysis showed that postoperative neck brace compared with no brace can relieve neck pain at 1 week, 2 weeks, 3 weeks, 6 weeks, 3 months, 12 months, and 24 months after surgery. The results of the meta-analysis are that −3.00 (−4.96 to −1.04) at the follow-up of 1 week after surgery, −2.70 (−4.90 to −0.50) at the follow-up of 2 weeks after surgery, −2.30 (−3.69 to −0.91) at the follow-up of 3 weeks after surgery, −0.98 (−1.59 to −0.37) at the follow-up of 6 weeks after surgery, −0.87 (−1.44 to −0.30) at the follow-up of 3 months after surgery −1.30 (−1.81 to −0.80) at the follow-up of 12 months after surgery, and −1.26 (−1.81 to −0.71) at the follow-up of 24 months after surgery. However, there was no significant difference between the 2 groups at the follow-up of 6 months (MD, 0.22; 95% CI, [−0.25 to 0.69]).

#### 3.3.2. Neck Disability Index.

**Figure 4.**
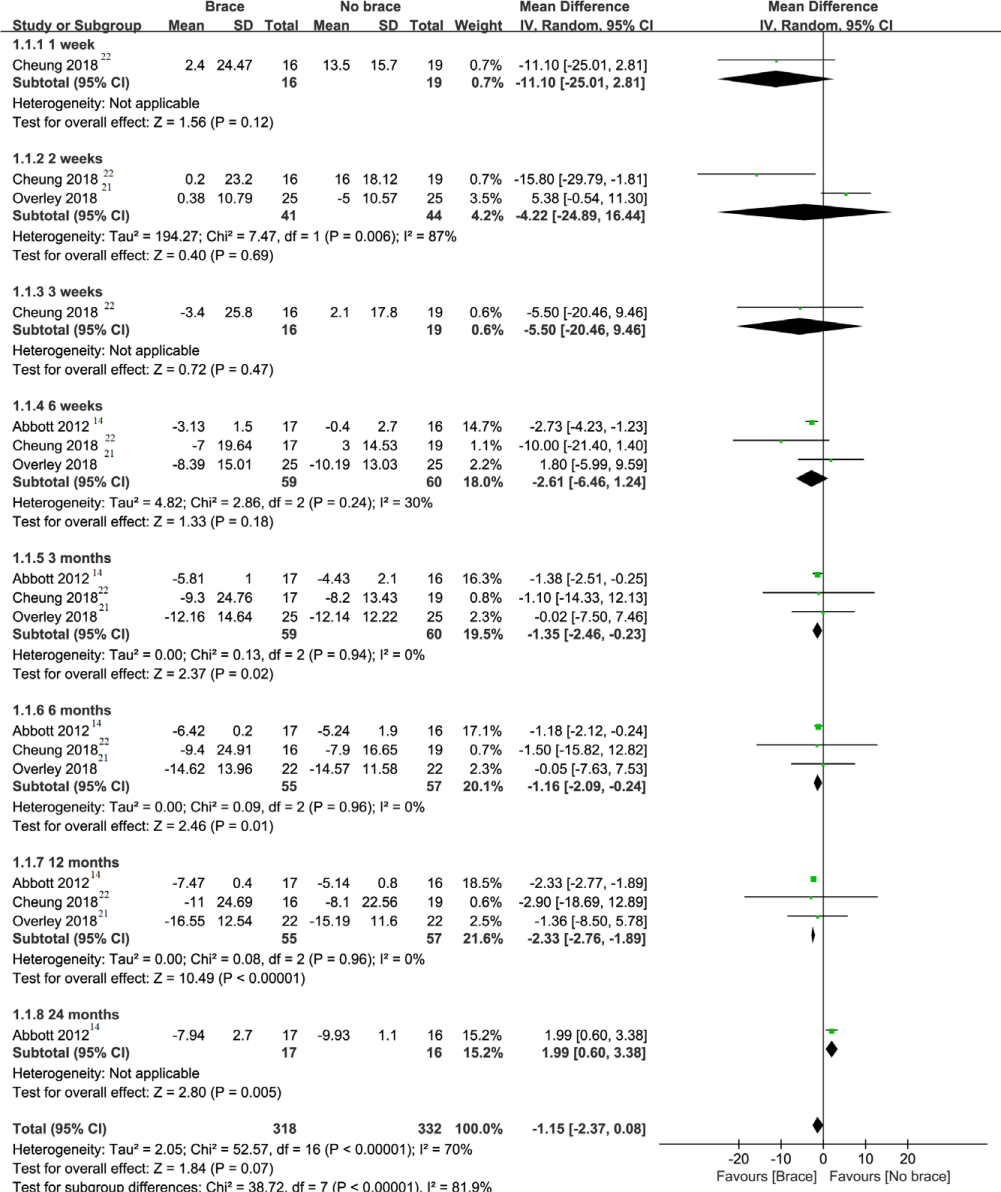
Forest plot and meta-analysis of NDI. CI = confidence interval, NDI = neck disability index, SD = standard deviation.

Three trials^[14,21,27]^ involving a total of 118 patients reported NDI as an outcome in the groups, as shown in Figure 4. Compared with the nonbrace group, the neck brace group had no statistical significance in terms of NDI within the follow-up of 6 weeks after surgery. The results of the meta- analysis are that −11.10 (−25.01 to 2.81) (MD and 95% CI) at the follow-up of 1 week after surgery, −4.22 (−24.89 to 16.44) at the follow-up of 2 weeks after surgery, −5.50 (−20.46 to 9.46) at the follow-up of 3 weeks after surgery, and −2.61 (−6.46 to 1.24) at the follow-up of 6 weeks after surgery. During the 3 to 12 month follow-up period, the NDI in the cervical brace group was slightly better than the nonbraced group (−1.35 [−2.46 to −0.23] at 3 months, −1.16 [−2.09 to −0.24] at 6 months, and −2.33 [−2.77 to −1.89] at 12 months). However, 1 study,^[14]^ only including 33 patients, reported the result of follow-up of 24 months, suggesting tend to get the opposite (1.99 [0.60–3.38]).

#### 3.3.3. SF-36 (Physical Component Summary scale).

**Figure 5.**
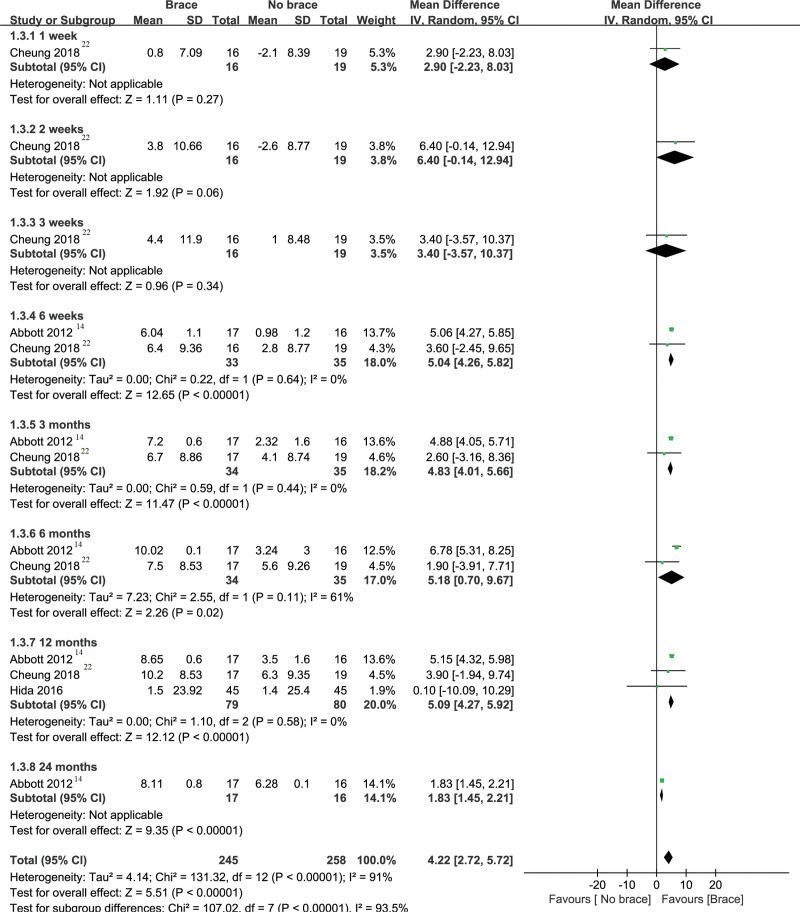
Forest plot and meta-analysis of SF-36 (PCS). CI = confidence interval, PCS = Physical Component Summary, SD = standard deviation, SF = 36-short form health survey.

Three studies^[14,20,27]^ involving a total of 158 patients showed the SF-36 Physical Component Summary scale (PCS) with cervical brace and nonbrace after cervical surgery (see Fig. 5). In the early 3 weeks after surgery, there was no significant difference between the cervical brace group and the nonbraced group (2.90 [−2.23 to 8.03] at 1 week, 6.40 [−0.14 to 12.94] at 2 weeks, and 3.40 [–3.57 to 10.37] at 3 weeks). During the 6 week to 24 month follow-up period, the SF-36 (PCS) in the cervical brace group was better than the nonbraced group (5.04 [4.26–5.82] at 6 weeks, 4.83 [4.01–5.66] at 3 months, 5.18 [0.70–9.67] at 6 months, 5.09 [4.27–5.92] at 12 months, and 1.83 [1.45–2.21] at 24 months).

#### 3.3.4. SF-36 (Mental Component Summary scale).

**Figure 6.**
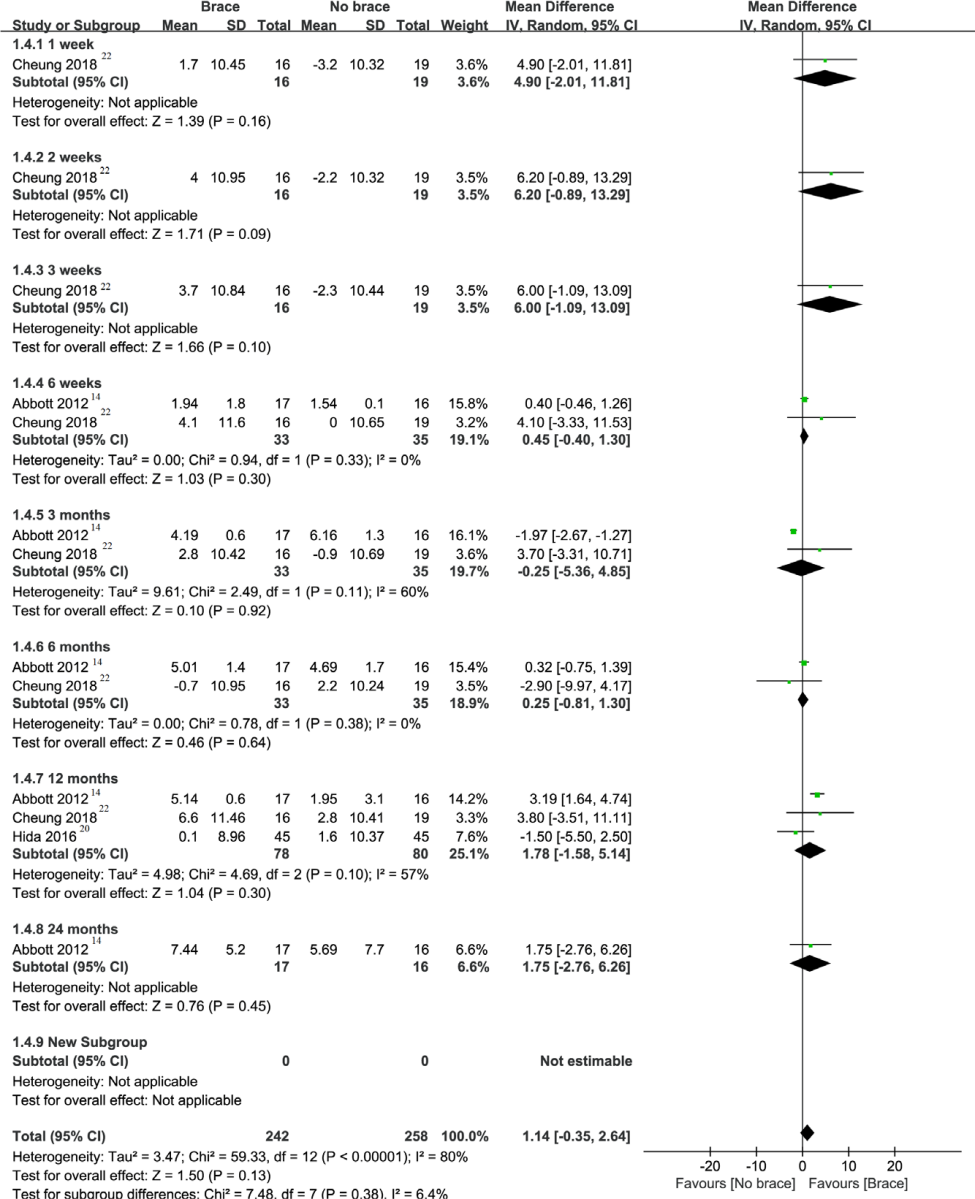
Forest plot and meta-analysis of SF-36 (MCS). CI = confidence interval, MCS = Mental Component Summary, SD = standard deviation, SF-36 = 36-short form health survey.

As shown in Figure 6, of the 4 RCTs reporting using brace after cervical surgery, 3 studies^[14,20,27]^ explicitly stated the SF-36 (Mental Component Summary scale [MCS] scale) result involving a total of 158 patients. According to different follow-up times, the results are that (MD and 95% CI) 4.90 (−2.01 to 11.81) at 1 week, 6.20 (−0.89 to 13.29) at 2 weeks, 6.00 (−1.09 to 13.09) at 3 weeks, 0.45 (−0.40 to 1.30) at 6 weeks, −0.25 (−5.36 to 4.85) at 3 months, 0.25(−0.81 to 1.30) at 6 months, 1.78 (−1.58 to 35.14) at 12 months, and 1.75 (−2.76 to 6.26) at 24 months. Therefore, SF-36 (MCS) does not change significantly in the brace group or the nonbrace group at any time after surgery.

#### 3.3.5. Grading of recommendations assessment, development, and evaluation.

The GRADE level of evidence is moderate for all of the results. Table 2 shows the GRADE evidence profiles. The main reason for the decreasing level was small sample size.

**Table 2:** The GRADE evidence of outcomes.

Outcomes	No. of participants (studies)	Certainty of the evidence (GRADE)
NDI follow-up: range 2 wk to 24 mo	112(3 RCTs)	⨁⨁⨁◯ MODERATE*
SF-36 (PCS) follow-up: range 2 wk to 24 mo	142(3 RCTs)	⨁⨁⨁◯ MODERATE*
Neck pain follow-up: range 1 wk to 24 mo	142(3 RCTs)	⨁⨁⨁◯ MODERATE*
SF-36 (MCS) follow-up: range 1 wk to 24 mo	142(3 RCTs)	⨁⨁⨁◯ MODERATE*

GRADE = Grading of Recommendations Assessment, Development, and Evaluation, MCS = Mental Component Summary, NDI = neck disability index, PCS = Physical Component Summary, RCT = randomized controlled trial, SF-36 = 36-short form health survey.

* Sample size is small.

#### 3.3.6. Publication bias.

For the same outcome, the number of included trials (<10) was too small to conduct any sufficient additional analysis of publication bias.

## 4. Discussion

To the best of our knowledge, this is the first meta-analysis that provides comprehensive and quantitative evidence for postoperative cervical spine with and without cervical brace. In addition, the purpose of this study was to examine the pain, functional outcome, and quality of life with the 2 methods of postoperative management. In this meta-analysis of 4 trials, 208 patients were included. Overall, we found that postoperative use of the cervical brace can relieve cervical pain slightly and partially improve NDI and SF-36 (PCS) and intermittently showed statistically significant improvements. However, for SF-36 (MCS), there was no significant difference between the braced group and the nonbraced group.

Postoperative cervical pain typified by persistent pain around the neck and shoulders is a common problem associated with cervical laminoplasty and anterior cervical decompression and fusion.^[28–30]^ Although the exact cause of axial neck pain has not been detected, intraoperative invasion to the cervical muscles is presumed to be involved in this complication.^[30–32]^ External cervical brace can limit cervical activity. The use of rigid cervical neck collars has been shown to reduce the usual cervical range of motion by 62.9%,^[33]^ which may promote the repair of neck tissue,^[34]^ so it is a possible cause of pain relief. In the present research, postoperative use of the cervical brace can relieve cervical pain compared with no neck brace during the 24-month follow-up period. Only in the 6 months after surgery, the braced group shows no advantage in relieving cervical pain. At the same time, the advantage of wearing a neck brace to relieve neck pain tends to decline over time(−3 for 1 week, −2.7 for 2 weeks, −2.3 for 3 weeks, −0.98 for 6 weeks, −0.87 for 3 weeks, −1.3 for 12 months, and −1.07 for 24 months). Furthermore, to evaluate treatment effectiveness, Carreon et al^[35]^ have reported the minimal clinical important difference (MCID) for pain to be 2.5. Therefore, the pain relief in only 2 weeks with and without neck brackets has clinical significance. The research by Ebata et al^[36]^ showed that the postoperative neck pain of patients wearing cervical brace scores significantly reduced at 2 weeks following cervical laminoplasty surgery, which is similar to our results. In addition, according to a questionnaire study sampling spine surgeons, 63% of spine surgeons employed routine postoperative cervical bracing following surgery may be due to improving pain relief.^[37]^

We used NDI to evaluate cervical function. Braced group for 3, 6, and 12 months could reduce the NDI, but there was reversed result for 24 months. Actually, only the study by Abbott et al^[14]^ in all the literature included in this meta-analysis showed that nonbraced group was slightly better in NDI than braced group at 24 months. In addition, the author reported more than 50% loss to follow-up in NDI outcome measures at 24 months. Therefore, this study outcome should be interpreted with caution. According to the study by Carreon et al,^[35]^ MCID for NDI is 7.5, so for the outcome of NDI in this study, although there are statistical differences in the improvement of cervical brace wearing after cervical surgery, there is no clinical significance.

With regard to the quality of life, both procedures were found to be associated with significant improvement in SF-36 (PCS) compared with baseline. Additional, we found a statistically significant difference of 5.04 points, 4.83 points, 5.18 points, 5.1 points, and 1.83 points for SF-36 (PCS) in favor of the braced group from 6 weeks to 24 months, clinical significance exists except for 24 months in line with the study by Carreon et al,^[35]^ and MCID for SF-36 (PCS) is 4.1. However, no association between postoperative cervical brace and SF-36 (MCS) was seen in our study. This study was able to identify that the use of cervical brace can be associated with the improvement of SF-36 (PCS) at 6 weeks after operation and may be related to the early cervical vertebral movement restriction, which is beneficial to the healing of the incision and the fusion of vertebral, so as to relieve the preoperative symptoms better. Similarly, in the study by Campbell et al,^[13]^ significant improvements in SF-36 (PCS) were seen at the follow-up period of 6 to 24 months for cervical braced group. SF-36 (MCS) is related to mental health, and there appears to be no significant change at all time intervals. However, the patients wearing neck brace in the first 3 weeks after operation have a tendency to improve SF-36 (MCS), which may be concerned with additional treatment of neck brace. The patients get some psychological comfort. From 6 weeks after the surgery, the neck brace was released during this time, and psychological factors were eliminated. Therefore, the improvement in the trend of SF-36 (MCS) has also disappeared.

There were some potential limitations. First, the test power was limited by sample size. Only 4 studies were included in this meta-analysis, and all of them had a relatively small sample size (n < 100) although all the literature from 8 databases is RCTs, which are considered as highly reliable and evidence-based study designs. Therefore, the GRADE shows a moderate level of evidence in all results, and the main reason for the downgrade is the small sample size. Second, the included studies in this meta-analysis were performed in different patient groups, different cervical surgical procedures, different braces, and various clinical settings. Therefore, the risk of introducing potential heterogeneity is present. Finally, publication bias will have influenced results to some degree, but the number of included trials (<10) for the same outcome was too small to conduct any sufficient additional analysis of publication bias. However, we strictly adhered to the Preferred Reporting Items for Systematic Reviews and Meta-analyses guidelines to improve the quality of this systematic review and meta-analysis.

## 5. Conclusions

The limited existing literature does seem to suggest that wearing cervical brace helps relieve pain. In addition, the improvement of cervical function by brace is mainly from 3 to 12 months after surgery. As for the quality of life, cervical brace is recommended to improve the state of physical health from 6 weeks to 24 months. However, there is no relevant evidence indicating whether cervical brace is an option for mental health of patients. Besides, the evidence of all results is moderate. More higher quality randomized studies are needed to verify the current conclusions.

## Author contributions

The study was conceptualized by ZHF, and MY, MY, and JDZ searched and selected the trials, and extracted, analyzed, and interpreted the data. MY drafted the article. ZHF and JDZ helped with the study design and critically reviewed the article. All authors read and approved the final version of the article.
